# The potential anti-arrhythmic effect of SGLT2 inhibitors

**DOI:** 10.1186/s12933-024-02312-0

**Published:** 2024-07-15

**Authors:** Hong-Yi Duan, Hector Barajas-Martinez, Charles Antzelevitch, Dan Hu

**Affiliations:** 1 Department of Cardiology and Cardiovascular Research Institute, Renmin Hospital of Wuhan University, 238 Jiefang Road, Wuhan, 430060 Hubei China; 2 Hubei Key Laboratory of Cardiology, Wuhan, 430060 Hubei China; 3 Lankenau Institute for Medical Research, Lankenau Heart Institute, Wynnewood, PA 19096 USA; 4 Sidney Kimmel Medical College, Thomas Jefferson University, Philadelphia, 19107 USA

**Keywords:** SGLT2 inhibitor, Arrhythmias, Intracellular sodium, Myocardial metabolism, Autophagy

## Abstract

Sodium-glucose cotransporter type 2 inhibitors (SGLT2i) were initially recommended as oral anti-diabetic drugs to treat type 2 diabetes (T2D), by inhibiting SGLT2 in proximal tubule and reduce renal reabsorption of sodium and glucose. While many clinical trials demonstrated the tremendous potential of SGLT2i for cardiovascular diseases. 2022 AHA/ACC/HFSA guideline first emphasized that SGLT2i were the only drug class that can cover the entire management of heart failure (HF) from prevention to treatment. Subsequently, the antiarrhythmic properties of SGLT2i have also attracted attention. Although there are currently no prospective studies specifically on the anti-arrhythmic effects of SGLT2i. We provide clues from clinical and fundamental researches to identify its antiarrhythmic effects, reviewing the evidences and mechanism for the SGLT2i antiarrhythmic effects and establishing a novel paradigm involving intracellular sodium, metabolism and autophagy to investigate the potential mechanisms of SGLT2i in mitigating arrhythmias.

**Graphical abstract:**

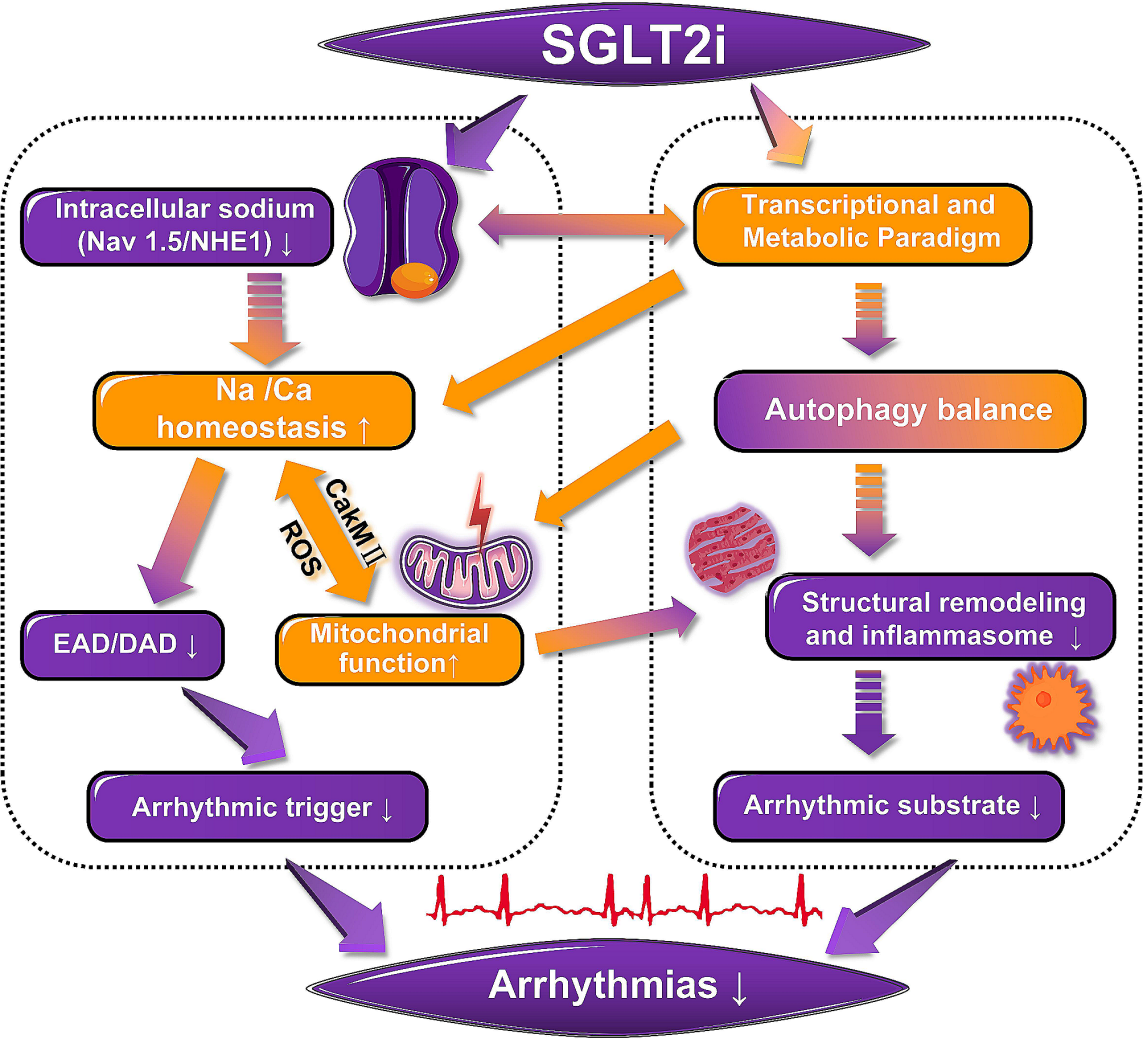

**Supplementary Information:**

The online version contains supplementary material available at 10.1186/s12933-024-02312-0.

## Introduction

Since 2008, The FDA’s regulatory guidance recommended all glucose-lowering therapies undergoing evaluation should rule out increased cardiovascular risk. There are numerous cardiovascular outcome studies have been conducted, but most have not shown cardiovascular benefits. Surprisingly, the EMPA-REG OUTCOME trial, a long-term, multicenter, randomized, double-blind, placebo-controlled clinical trial, became the beginning of the cardiovascular application of SGLT2i. This study showed that treatment with the SGLT2i (empagliflozin) in diabetic patients with cardiovascular high-risk factors significantly reduced the incidence of major combined cardiovascular outcome and all-cause mortality, as compared to placebo, over a median follow-up of 3.1 years. Although increased risks of genital infections were shown. The risk of serious adverse events including diabetic ketosis was not significantly increased compared with placebo, demonstrating the exciting cardiovascular potential and safety of SGLT2i [1].

**Fig. 1:**
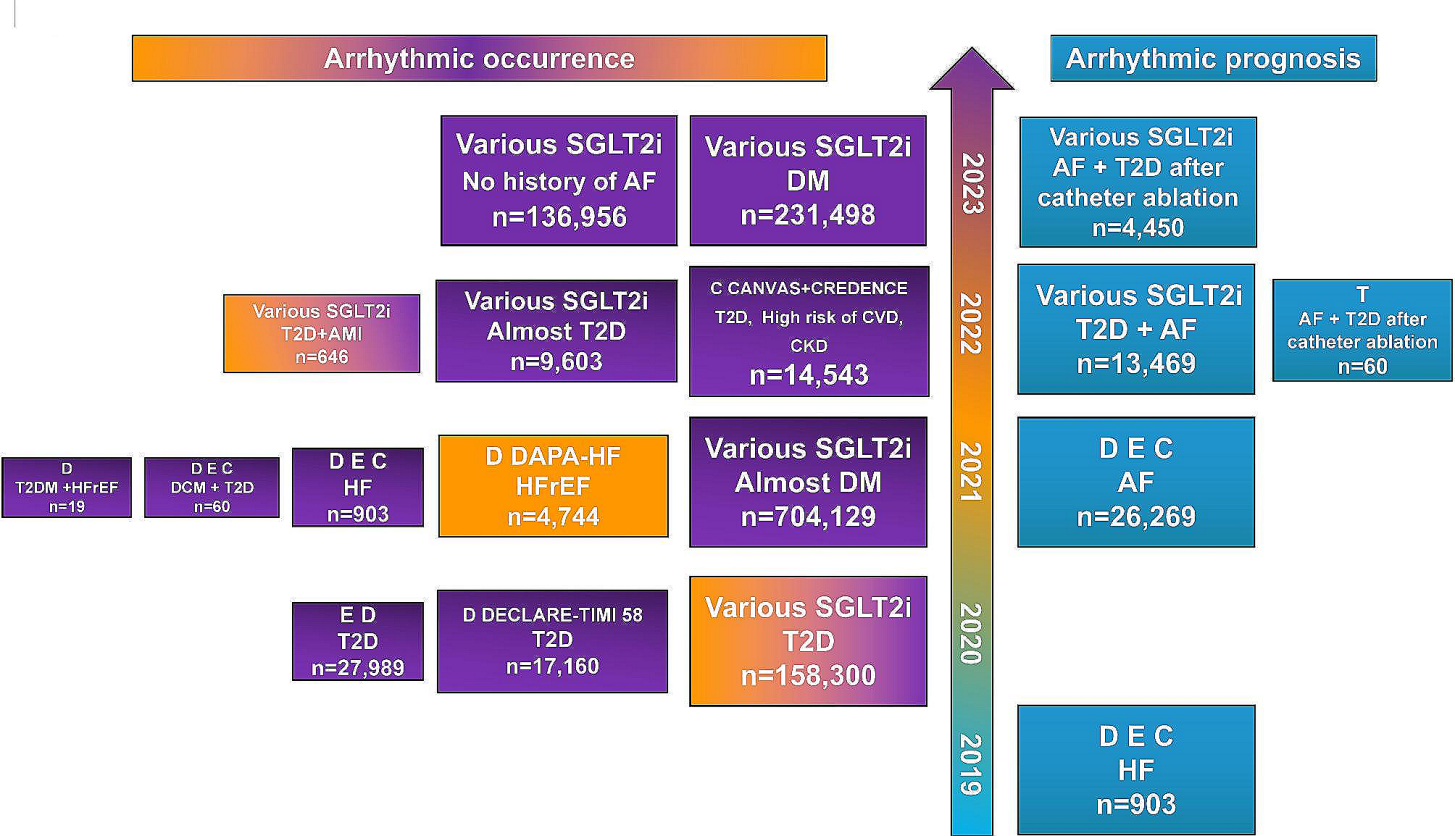
Clinical research on the effect of SGLT2i on arrhythmia in the past four years. Purple indicates a decrease in atrial arrhythmia, orange indicates a decrease in ventricular arrhythmia, both colors indicate a decrease in both atrial and ventricular arrhythmia, and blue indicates an improvement in arrhythmia prognosis. *D* Dapagliflozin, *E* Empagliflozin, *C* Canagliflozin, *T* Tofogliflozin, *DCM* dilated cardiomyopathy, *CVD* cardiovascular disease, *AMI* acute myocardial infarction, *T2D* type 2 diabetes, *DM* diabetes mellitus, *AF* atrial fibrillation, *HF* heart failure, *HFrEF* heart failure with reduced ejection fraction, *DAPA-HF* dapagliflozin and prevention of adverse outcomes in heart failure, *DECLARE-TIMI58* dapagliflozin effect on cardiovascular events-thrombolysis in myocardial infarction 58, *CANVAS* canagliflozin cardiovascular assessment study, *CREDENCE* canagliflozin and renal events in diabetes with established nephropathy clinical evaluation

Subsequently, abundant clinical trials and meta-analysis convinced people to identify SGLT2i application against cardiovascular outcomes and deaths, especially in HF [2, 3]. 2021 ESC Guidelines recommend SGLT2i for HF patients with reduced ejection fraction [4]. The latest meta-analysis supported SGLT2i’s role as a foundational therapy for HF, which reduced the risk of cardiovascular death and hospitalizations for patients with HF regardless of the ejection fraction [2]. 2022 AHA/ACC/HFSA Guideline expanded the application of SGLT2i to all stages of HF as a primary recommended medication [5]. Subsequently, the 2023 ESC guidelines also updated the same opinion and SGLT2i became the Class Ia recommended medication for patients with chronic heart failure [6]. It is worth mentioning that the mortality rate of cardiac sudden death in heart failure has decreased in the past decade, which can be attributed to the continuous improvement of evidence-based medications with antiarrhythmic effects [7]. Exactly, the promising data have emerged on the antiarrhythmic effects of SGLT2i recently (Fig. 1). Although most of these are from retrospective studies and subgroup analyses of related researches, where there are no large sample prospective researches on the antiarrhythmic actions of SGLT2i. Analysis from a global federated electronic medical record database suggested that various SGLT2i alleviated the occurrence of AF, all-cause death and lower the risk of composite of incident ventricular tachycardia/ventricular fibrillation (VT/VF) and cardiac arrest [8]. A population-based cohort study utilizing Taiwan’s National Health Insurance Research Database also obtained the result that SGLT2i decreased various kinds of new-onset arrhythmia including atrial fibrillation (AF), supraventricular arrhythmia, and ventricular arrhythmias (VAs) [9]. Similarly, less occurrence of total cardiac arrhythmia with various SGLT2i treatment was showed in a retrospective study included patients diagnosed with T2D or controlled hypertension who prescribed the indicated glucose-lowering agents [10]. A meta-analysis, incorporating the population of diabetes mellitus (DM), chronic kidney disease (CKD), and HF, provided stronger evidence, demonstrated that SGLT2i were associated with a lower risk of AF, atrial flutter (AFL) and VT [11] (Supplemental Table S1). Meanwhile, SGLT2i were found to improve electrocardiography indices and cardiac electromechanics in DM patients, and decrease arrhythmia in mode animals with HF and cardiac ischemia/reperfusion injury [12–14]. Notedly, SGLT2i do not affect the electrocardiogram of patients with normal heart rhythm [15]. These findings suggest that SGLT2i hold promise as a potential new antiarrhythmic drug from prevention to treatment.

## SGLT2i in atrial arrhythmias

A meta-analysis of 34 randomized controlled trials including all trials related SGLT2i until December 2020 suggested SGLT2i potential against atrial arrhythmias, simultaneously decreasing cardiac death [16] (Supplemental Table S1). Some clinical studies have found the SGLT2i could improve outcomes in patients with AF, similar to its effect in HF patients (Fig. 1; Table 1). Data from the EMPA-REG OUTCOME trial showed that Empagliflozin decrease the CV death and HF hospitalization in patients with AF and without AF [17]. Analyses of Korean health databases showed that SGLT2i treatment helped patients with T2D and AF achieved lower rates of hospitalization for HF and all-cause mortality [18]. This finding was supported by a meta-analysis of data extracted from 3 RCTs with 2 sotagliflozin (dual SGLT1/2 inhibitor) and 1 Empagliflozin suggesting net protection by the SGLT2i against cardiovascular outcomes and deaths in patients with T2D and AF [19]. Considering AF separately, a retrospective study with a global medical research network database demonstrated that dapagliflozin, empagliflozin and canaglifozin decreased cardioversion and all-cause mortality in patients with AF [20]. Importantly, SGLT2i also have beneficial effects in AF patients with T2D who have undergone catheter ablation (CA). A randomized controlled study involving eighty patients demonstrated that tofogliflozin is more effective than anagliptin in inhibiting AF recurrence after CA in T2D patients [21]. Subsequently, a study conducted using the TriNetX research network also yielded similar results, showing that the use of SGLT2i in AF patients with T2D can reduce the risk of arrhythmia recurrence following CA [22].

**Table 1:** Clinical studies on the improvement of arrhythmia prognosis with SGLT2i

Drug	No. of Patients			Population	Main findings			References
Tofogliflozin	38 SGLT2i	32 DPP4i		AF + T2D after catheter ablation	Recurrent AF ↓ (24% VS 48%, *P* = 0.042)			[21]
Various SGLT2i	2225 SGLT2i	2225 no-SGLT2i		AF + T2D after catheter ablation	Cardioversion ↓ (OR 0.62) new AAD therapy ↓ (OR 0.72) re-do AF ablation ↓ (OR 0.71) HF exacerbations ↓ (OR 0.81) All-cause hospitalization ↓ (OR 0.65) All-cause mortality ↓(OR 0.62) Event-free survival ↑ (HR 0.85)			[22]
Empagliflozin	389 T2D with AF	6631 T2D without AF		T2D ± AF	CV death or HF hospitalization ↓ in patients with AF (HR 0.58) and without AF (HR 0.67)			[25]
Dapagliflozin, Empagliflozin Canaglifozin	26,294 SGLT2i	1,368,518 no-SGLT2i		AF	Cardioversion ↓ (HR 0.92) All-cause mortality ↓ (HR 0.68) Ischemic stroke ↑ (HR 1.08)			[20]
Various SGLT2i	2958 SGLT2i	10,691 no-SGLT2i		T2D + AF	Hospitalization for HF ↓ (HR 0.70) All-cause mortality ↓ (HR 0.74)			[18]

*SGLT2i* sodium-glucose co-transporter 2 inhibitors, *RCT* randomized controlled trial, *T2D* type 2 diabetes, *DM* diabetes mellitus, *AF* atrial fibrillation, *AFL* atrial flutter, *CV* cardiovascular, *SCD* sudden cardiac death, *AAD* antiarrhythmic drug, *OR* odds ratio, *HR* hazard ratio, *RR* relative risk

Not only for improving prognosis of AF patients, several clinical analyses and trials have found that SGLT2i reduced the incidence of AF in diabetic patients and HF patients (Fig. 1; Table 2). A similar result was also obtained in T2D patients with acute myocardial infarction (AMI) [23]. A large sample analysis from the Food and Drug Administration adverse event reporting system demonstrated a significant decrease in the incidence of AF. These reports are almost from diabetes mellitus patients [24]. And the finding was also identified by several meta-analysis [11, 19]. Moreover, as for other population except DM patients, a meta-analysis of eight trials comprising patients with or without DM confirmed the hypothesis that SGLT2i decrease the incidence of AF. Subgroup analysis showed that fewer AF incidents occurred with follow-up of more than one year, in patients utilizing dapagliflozin, and in patients with a history of cardiovascular disease or cardiovascular risk factors [26]. In addition, another meta-analysis showed similar result that various SGLT2i could reduce the incidence of AF in patients with or without DM [27]. Meanwhile SGLT2i were found to reduce the incidence of new-onset AF in T2D patients and T2D patients with other cardiovascular diseases, such as AMI or non-ischemic DCM [9, 23, 28]. In addition, a recent mendelian randomization study also revealed that genetically SGLT2i was associated with reduced risk of T2DM and AF [29].

**Table 2:** Clinical studies on the antiarrhythmic action of SGLT2i in atrial arrhythmias

Drug	No. of Patients		Population	Main findings		References
Dapagliflozin Empagliflozin Canaglifozin	32 SGLT2i	28 no-SGLT2i	Non-ischemic DCM + T2D	New-onset AF ↓ (log-rank *p* = 0.04)		[30]
Empagliflozin Dapagliflozin	15,606 SGLT2i	12,383 DPP4i	T2D	New-onset AF ↓ (HR 0.61)		[28]
Various SGLT2i	79,343 SGLT2i	57,613 GLP-1	No history of AF	new-onset AF ↓ (aHR: 0.87)		[31]
Various SGLT2i	79,150 SGLT2i	79,150 placebos	Newly diagnosed with T2D	All-cause mortality ↓ (aHR 0.55) New-Onset arrhythmias ↓ (aHR 0.83) including AF, supraventricular arrhythmias, and VAs		[9]
Various SGLT2i	111 SGLT2i	535 no-SGLT2i	T2D + AMI	New-onset cardiac arrhythmias ↓ (6.3 vs.15.7%) AF ↓ and VT/VF ↓ (p 0.032)		[23]
Dapagliflozin Empagliflozin Canagliflozin	903 patients		HF	AF ↓ (OR 0.76)		[32]
Various SGLT2i	62,098 SGLT2i	642,031 controls	Almost DM	AF ↓ (PRR 0.55)		[24]
Various SGLT2i	3203 SGLT2i	6406 no SGLT2i	T2D or prescribed the glucose-lowering agents for controlling hypertension	Total cardiac arrhythmia ↓ (HR 0.58) AF ↓ (HR 0.56) Stroke ↓ (HR 0.48), HF ↓ (HR 0.54) MI ↓ (HR 0.47)		[10]
Various SGLT2i	115,749 SGLT2i	115,749 no SGLT2i	DM	HF ↓ (HR 0.70) All-cause mortality ↓ (HR 0.61) Cardiac arrest ↓ (HR 0.70) AF ↓ (HR 0.81) Ischemic stroke/TIA ↓ (HR 0.90) Composite of VT/VF and cardiac arrest ↓ (HR 0.76)		[8]

*SGLT2i* sodium-glucose co-transporter 2 inhibitors, *RCT* randomized controlled trial, *T2D* type 2 diabetes, *DM* diabetes mellitus, *AF* atrial fibrillation, *AFL* atrial flutter, *VA* ventricular arrhythmia, *HF* heart failure, *AMI* acute myocardial infarction, *CKD* chronic kidney disease, *HFrEF* heart failure with reduced ejection fraction, *TIA* transient ischemic attack, *SCD* sudden cardiac death, *OR* odds ratio, *HR* hazard ratio, *RR* relative risk, *aHR* adjusted hazard ratio, *pOR* partial odds ratio

Insights from the DECLARE-TIMI 58 trial suggested that SGLT2i could reduce the occurrence of AF/AFL in T2D patients regardless of the history of AF/AFL [33]. This finding was supported by a meta-analysis of 16 randomized controlled trials in T2D patients that various SGLT2i treatment reduced the occurrence of AF/AFL and all-cause mortality compared to placebo [34]. A secondary analysis from the CANVAS program and CREDENCE trial demonstrated that canagliflozin could reduce the incidence of AF/AFL in participants with no AF/AFL history and alleviated AF/AFL-related complications [35]. Another meta-analysis of 22 RCTs in T2D or HF patients showed that SGLT2i treatment had less AF/AFL, arrhythmia and intracardiac thrombosis. Subgroup analysis of this research suggested less AF/AFL incidence with SGLT2i treatment in women or dapagliflozin treatment [36]. It is worth noting that based on the meta-analysis discussed above, dapagliflozin may have a more significant impact on reducing the incidence of atrial arrhythmias [26, 36]. However, it should be acknowledged that there are no studies specifically designed to explore this assumption. Therefore, further research is needed to confirm whether dapagliflozin is more effective than other SGLT2i in reducing the risk of atrial arrhythmias, and to determine the mechanisms underlying its potential beneficial effects.

## SGLT2i in ventricular arrhythmias

Sudden cardiac death (SCD) due to an arrhythmogenic event is a common terminal event in HF, with VAs being the frequent cause. Given that the studies discussed herein about SCD and all-cause mortality, it is reasonable to speculate that SGLT2i may play an active role in reducing the incidence of Vas (Fig. 1; Table 3). There are several researches supporting this conception. Analysis from a global federated electronic medical record database demonstrated that SGLT2i reduced the occurrence of composite of VT/VF and cardiac arrest [8]. The data from DAPA-HF suggested that dapagliflozin decreased the occurrence of serious VAs, resuscitated cardiac arrest, and sudden death in HF patients with reduced ejection fraction (HFrEF) [37]. Analogously in a small double-blind, crossover, placebo-controlled trial, the decrease in ventricular ectopy was identified in 2-week treatment with dapagliflozin in T2D patients with HFrEF [38]. In T2D patients with AMI, it was demonstrated that the reduction of new-onset cardiac arrhythmias and VT/VF with various SGLT2i treatment [23]. As mentioned above, this conception was also supported by the meta-analysis form 22 RCTs, which showed SGLT2i treatment decreased the VT incident compared to placebo [11]. Interestingly, there is a meta-analysis comprising 19 RCTs that low-dosage SGLT2i therapy but not high-dosage SGLT2i therapy reduced the occurrence of VAs compared to control or placebo. The population of this analysis included patients with T2D and/or HF and/or CKD. This insight is different from the results of SGLT2i therapy in cardiovascular morbidity and mortality or atrial arrhythmia that there is no distinction between high-dose and low dose for SGLT2i treatment [1, 39]. This discrepancy may imply a unique anti-ventricular arrhythmias mechanism of SGLT2i.

**Table 3:** Clinical studies on the antiarrhythmic action of SGLT2i in ventricular arrhythmias

Drug	No. of Patients		Population	Main findings		References
Various SGLT2i	79,150 SGLT2i	79,150 placebos	Newly diagnosed with T2D	All-cause mortality ↓ (aHR 0.55) New-Onset arrhythmias ↓ (aHR 0.83) including AF, supraventricular arrhythmias, and VAs		[9]
Dapagliflozin	2373 dapagliflozin	2371 placebo	HFrEF	Serious VAs, resuscitated cardiac arrest, or sudden death ↓ (HR 0.79 )		[37]
Dapagliflozin	19 patients		T2D + HFrEF	2 week treatment with dapagliflozin, ventricular ectopy ↓ (1.4% vs. 0.2%)		[38]
Various SGLT2i	115,749 SGLT2i	115,749 no-SGLT2i	DM	All-cause mortality ↓ (HR 0.61) Cardiac arrest ↓ (HR 0.70) Composite of VT/VF and cardiac arrest ↓ (HR 0.76)		[8]
Various SGLT2i	111 SGLT2i	535 no-SGLT2i	T2D + AMI	New-onset cardiac arrhythmias ↓ (6.3 vs.15.7%) AF ↓ and VT/VF ↓ (p 0.032)		[23]

*SGLT2i* sodium-glucose co-transporter 2 inhibitors, *RCT* randomized controlled trial, *T2D* type 2 diabetes, *DM* diabetes mellitus, *AF* atrial fibrillation, *AFL* atrial flutter, *CV* cardiovascular, *VA* ventricular arrhythmia, *VT* ventricular tachycardia, *VF* ventricular fibrillation, *HF* heart failure, *CKD* chronic kidney disease, *HFrEF* heart failure with reduced ejection fraction, *OR* odds ratio, *HR* hazard ratio, *RR* relative risk, *aHR* adjusted hazard ratio

## Antiarrhythmic mechanisms

**Fig. 2:**
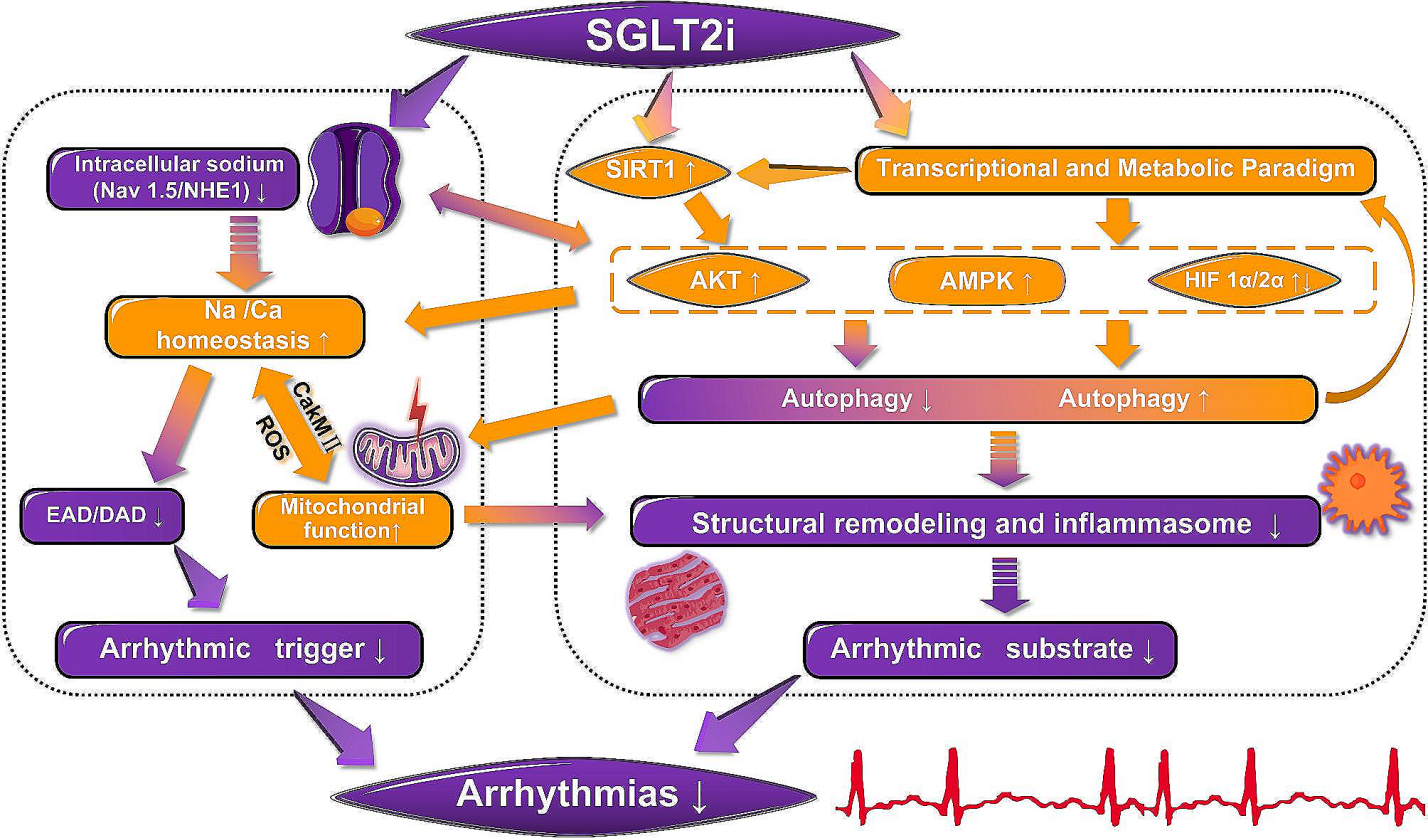
Possible mechanism diagram of SGLT2i therapy for arrhythmia. SGLT2i impacts intracellular sodium levels through its actions on Nav1.5 (regulating INa,L) and NHE1. Meanwhile, it evokes a transcriptional and metabolic paradigm resembling hypoxia and fasting, and optimizes autophagy with the reduction in structural remodeling and inflammation, accompanied by alterations in multiple molecular entities such as AMPK, SIRT1, HIF, and AKT. These two pathways complement and cooperate with each other to promote the antiarrhythmic effects of SGLT2i via decreasing arrhythmic trigger and substrate. *Nav1.5* voltage-gated sodium channel 1.5, *INa,L* late sodium current, *NHE1* sodium-hydrogen exchanger 1, *AMPK* AMP-activated protein kinase, *SIRT1* sirtuin 1, *HIF* hypoxia-inducible factor, *AKT* protein kinase B, *EAD* early afterdepolarization, *DAD* Delayed afterdepolarization, *CakM II* calcium/calmodulin-dependent protein kinase II, *ROS* reactive oxygen species

The cardiovascular protective effects of SGLT2i in no DM patients [32] suggests the protective mechanisms that are independent of improved glycaemic control and adds further evidence to the emerging concept that the SGLT2i possesses a direct protective effect on the heart. There are six different Sodium-glucose cotransporter isoforms have been reported, and two transporters, SGLT1 and SGLT2 proteins, have been widely studied. The SGLT1 is found in cardiac capillaries and cell membrane of cardiomyocytes in humans, suggested to be involved in glucose transport from capillaries into the cardiomyocytes. Conversely, SGLT2 protein is can’t be detected in the heart [40]. There is different selectivity for inhibition of SGLT2 vs. SGLT1 between various SGLT2i. Dapagliflozin, empagliflozin, luseogliflozin and tofogliflozin have high SGLT2/SGLT1 selectivity (≥ 1000 fold), whereas the selectivity of canagliflozin and ipragliflozin is lower (190 and 250 fold, respectively) [41]. And sotagliflozin is dual SGLT1/2 inhibitor. Therefore, the direct cardiovascular protective effects are unlikely achieved through SGLT2 suppression. The greater possibility is that the unexpected off-target actions of various SGLT2i directly or indirectly impact on the cardiomyocyte as a “class effect” considered that they are structurally related as derivatives of phlorizin. Currently, investigated possible targets focus on cardiac sodium-hydrogen exchanger 1 (NHE1) and late sodium channel current (INa,L), which could reduce intracellular sodium and calcium overload subsequently causing a chain of reaction, such as reduction in abnormal triggers, decreasing calmodulin-dependent kinase II (CaMKII) activity, cellular stress, inflammasome activation, fibrosis and so on. However, in some studies, the cardioprotective effect of SGLT2i was found to be independent of NHE1 [42, 43]. Importantly, Packer et al. put forward altering in metabolism induced by SGLT2i treatment causes the hypoxia and fasting-like transcriptional and metabolic paradigm, that may represent another key mechanism by which myocardial and mitochondrial function can be improved, and oxygen stress, inflammation and fibrosis can be alleviated through the regulation of autophagy via a series of molecular changes [44, 45]. Furthermore, it can be speculated that SGLT2i treatment has the potential to improve the substrate and re-entry mechanisms of arrhythmias. These effects can, in turn, lead to alterations in metabolic markers, generating a positive feedback loop. We make hypothesis that these two pathways (intracellular sodium, and transcriptional and metabolic changes) together explain the antiarrhythmic effect of SGLT2i. They play a crucial part in the arrhythmic substrates and triggers modified by SGLT2i, that can complement and cross each other (Fig. 2).

### Intracellular sodium

Na^+^ enters the cardiac muscle cell primarily through INa, Na–Ca exchange, and Na–H exchange [46]. Increased intracellular sodium is well known to result in an increase of intracellular Ca, via inhibiting the forward mode of Na/Ca exchange mechanism in the cell membrane of cardiac muscle cells. Na^+^ can also be ingested into mitochondria in exchange for Ca^2+^ via the mitochondrial Na^+^/Ca^2+^ exchanger both causing the cytosolic Ca^2+^ accumulation and disturbing the function of mitochondria to generate ATP and deploy antioxidant defense mechanisms [47]. Importantly, increased intracellular Ca^2+^ can contribute to arrhythmia by the arrhythmic substrates and triggers. A rise of intracellular Ca^2+^ can induce delayed afterdepolarization (DAD), early afterdepolarizations (EAD), arrhythmogenic transient inward current and spontaneous action potentials, while causing impaired relaxation or decreased diastolic function [48]. Meanwhile, Na-driven cytosolic and mitochondrial Ca^2+^overload triggers adverse effects of cardiac muscle. As a cellular messenger, Ca^2+^ also modulates the activity of various ion channels and signal paths, influencing the arrhythmic substrates (e.g. fibrosis and remodeling). All of these mechanisms might be involved in causing arrhythmia. Recently, NHE1 and INa,L were reported to be potential targets for reducing intracellular sodium for the favorable CV and antiarrhythmic actions of SGLT2i as drug class effect (Table 4). For instance, empagliflozin significantly altered INa,L, Na/hydrogen-exchanger currents, Ca^2+^ regulation and electrophysiological characteristics in DM cardiomyopathy, which supported the hypothesis that SGLT2i reduce intracellular sodium to relieve arrhythmia [49]. However, Paasche et al.’s latest research suggests that peak sodium current (INa,P) also participates the anti-arrhythmic effects of SGLT2i. SGLT2i can directly inhibit INa,P, altering atrial cardiomyocyte excitability [50]. The inhibition of SGLT1 has been studied as a potential mechanism for SGLT2i in reducing intracellular sodium. However, the relatively weak cross-over inhibition of SGLT1 observed with various SGLT2i and the lack of absolute evidence make this possibility less plausible [41]. Interestingly, although the inhibition of SGLT1 led to a decrease in myocardial reactive oxygen species (ROS) levels and enhanced phosphorylation of connexin-43, Lee et al. observed that dapagliflozin showed no inhibition of SGLT1 in a rat model of myocardial infarction. On the contrary, dapagliflozin upregulated the expression of SGLT1 by activating AMP-activated protein kinase (AMPK) [51].

**Table 4:** SGLT2i on intracellular sodium and calcium handling consequences with potential effects on arrhythmia mechanisms

Drug	Model	Ion related changes	Arrhythmia-related changes	References	
Empagliflozin	Streptozotocin-induced diabetic rats	INa, L ↓ Na/hydrogen-exchanger currents ↓ L-type Ca^2+^ currents ↑ Na^+^-Ca^2+^exchanger currents ↑ Incidence and frequency of Ca^2+^ sparks ↓ Sarcoplasmic reticular Ca^2+^ contents ↑ Intracellular Ca^2+^ transients ↑	QT intervals ↓ APD ↓		[49]
Dapagliflozin	Isolated atrial cardiomyocytes	INa, P↓	AP inducibility, amplitude and maximum upstroke velocity ↓		[50]
Dapagliflozin	Porcine large animal model	INa, P↓	Atrial-dominant reduction of myocardial conduction velocity		[50]
Empagliflozin	Cardiomyocytes from mice with heart failure and in cardiac Nav1.5 sodium channels containing the long QT syndrome 3 mutations	INa, L↓			[52]
Dapagliflozin Empagliflozin Canagliflozin	Cells expressing the recombinant human Nav1.5 α-subunit induced by H2O2	INa, L↓			[52]
Empagliflozin	Cardiomyocytes isolated from healthy mice, where INa, L was induced with veratridine		Spontaneous calcium transients ↓		[52]
Dapagliflozin Empagliflozin Canagliflozin	Mouse cardiomyocytes	NHE activity ↓			[53]
Dapagliflozin	H9c2 cells exposed to glucose deprivation for 24 h	NHE activity↓	Excessive autophagy ↓		[54]
Dapagliflozin	Rat model of pulmonary arterial hypertension-induced right heart failure via monocrotaline	Expression of key Ca^2+^ handling proteins ↑ Threshold for Ca^2+^ ↑ Susceptibility to spontaneous Ca^2+^ events ↓ Cellular Ca^2+^ handling ↑	Cx43 remodeling ↓ Conduction velocity ↑ Action potential duration alternans ↑ Susceptibility to spatially discordant APD alternans ↓ VA vulnerability ↓		[14]
Empagliflozin	Mouse myocyte with transverse aortic constriction exposed to empagliflozin 24 h	CaMKII activity ↓ Ca^2+^ spark frequency ↓ Sarcoplasmic reticulum Ca^2+^ load ↑ Ca^2+^ transient amplitude ↑			[55]
Empagliflozin	Human failing ventricular myocyte exposed to empagliflozin 30 min	Subsarcolemmal [Na+]i ↓ CaMKII activity no change Ca^2+^ handling no change			[55]
Empagliflozin	Human failing ventricular myocyte exposed to empagliflozin 24 h	Ca^2+^ spark frequency ↓ Ca^2+^ transient amplitude ↑			[55]

*INa, L* late sodium channel current, *INa, P* peak sodium current, *NHE* sodium/hydrogen exchanger, *AP* action potential, *APD* action potential duration, *Cx43* connexin 43

Myocardial sodium channel current that persists after INa,P is referred to INa,L. Although INa,L is relatively small compared to INa, P amplitude, it occurs throughout the low conductance phase of the action potential. Thus, it has a significant effect on the shape and duration of the action potential and the myocardial intracellular sodium loading [56]. Pathological enhance in INa,L can be observed in various hereditary and acquired arrhythmias including the long QT syndrome 3 and AF. In the presence of substrates that maintain arrhythmia (such as shortened APD, action potential duration, or fibrosis), INa,L enhancement may increase susceptibility to arrhythmia triggering mechanisms [57, 58]. Atrial myopathy is a significant concept that anatomical or structural changes are caused from AF, meanwhile provide substrates that maintain AF (AF begets AF) [59, 60]. By establishing a mathematical research model, Onal et al. proved that the increase of INa,L promoted the accumulation of Na^+^ and Ca^2+^ in atrial myocytes, which further caused the imbalance of intracellular Na^+^ and Ca^2+^ and accelerated the occurrence of AF [61]. Currently, Light et al. demonstrated that INa,L is one of the major targets for the antiarrhythmic effect of SGLT2i, increasing in cardiomyocytes from mice with HF induced by transverse aortic coarctation and in cardiac Nav1.5 sodium channels containing the long QT syndrome 3 mutations, and SGLT2i (dapagliflozin, empagliflozin and canagliflozin) inhibited INa,L in a concentration-dependent manner, with little inhibitory effect on INa,P. Further they found that SGLT2i could rapidly and reversely reduces the incidence of spontaneous calcium transients induced by INa,L agonists, and identified the decrease of cardiac nuclear-binding domain-like receptor 3 (NLRP3) inflammasome activation in an acute model of myocardial injury. Subsequently, the possibility of the binding sites (the amino acids F1760 and W1345 in the DIII-DIV site) for empagliflozin within Nav1.5 were exhibited using computer models [52]. F1760 was showed to be the binding site of ranolazine, a reported selective INa,L inhibitor [62].

The action of reducing intracellular sodium by SGLT2i intervene has also been partly ascribed to a decreased activity of the sodium–hydrogen exchanger (NHE), a family of exchangers that consist of structurally related isoforms mediating the exchange of sodium for hydrogen ions across cell membranes [53, 63]. NHE1 predominates in the heart, while NHE3 express restrictedly in the apical surface of renal and gastrointestinal epithelial cells [64]. In renal, NHE3 is responsible for a majority of the sodium re-uptake after glomerular filtration [64]. SGLT2 and NHE3 are functionally intertwined and co-localized. Inhibit SGLT2i could decrease the activity of NHE3 [65]. Contrarily, knockout of NHE3 depresses the expression of SGLT2 [66]. Some trial and experiment data suggest that the renal benefits effect of SGLT2i can be partially attribute to decreased intracellular sodium with NHE3 inhibition [67]. Therefore, the possibility exists that SGLT2i inhibit cardiac NHE1 with similar mechanism to reduce intracellular sodium explaining the cardioprotective effects. Activation of cardiac NHE1 in HF and diabetes has been reported as the potential association between the identified therapeutic effects of SGLT2i in the two diseases [68]. In addition, enhanced NHE1 has also been found in ischemia/reperfusion, cardiac hypertrophy and arrhythmias [69]. It is considered a possible common path in various stresses that could cause myocardium structural and functional damage, as well as ionic and electrophysiological remodeling [70–72]. The favorable effects of NHE1 inhibitors in HF has been identified [71–73]. And there are evidences supporting that inhibiting NHE1 might reduce the induction of cardiac arrhythmia. In rabbit hearts with hypertrophy and HF induced by pressure/volume overload, two-month NHE1 inhibitor treatment reduced duration of action potential QT-interval and QRS, restored sodium and calcium handling and alleviated the incidence of calcium after-transients, as well as hypertrophy and HF [74]. Another study found that treatment with NHE1 inhibitor cariporide prevented action potential prolongation and reduced the propensity to develop DAD. Similarly, NHE induced arrhythmogenic action of kappa-opioid receptor stimulation, and NHE blocker could significantly attenuate the induction of arrhythmia [75]. Of note, in animal models of VF, myocardial effects elicited by NHE1 inhibitors reducing Na and Ca overload, have been reported, such as preservation of left ventricular distensibility, return of cardiac activity with greater electrical stability, reduction of post-resuscitation episodes of VF and post-resuscitation myocardial dysfunction [76]. All these can support the antiarrhythmic effect of inhibiting NHE1 to reduce intracellular sodium. The computer simulation studies showed that empagliflozin, dapagliflozin and canagliflozin have high binding affinity with the extracellular Na-binding site of NHE [53]. Analogously, Xiang et al. also reveal the possibility that various SGLT2i bind to a structural model of NHE1via silico analysis in their research [54]. In summary, we can currently explore the possibility of anti-arrhythmic action of SGLT2i via nhe1 through these clues. It is a pity that the integrated pathway from SGLT2i to NHE1 to arrhythmia has not been investigated.

SGLT2i modulates calcium homeostasis and key calcium-handling proteins, including the reduction of CaMKII and RyR2 and the preservation of SERCA2a and Cav1.2 [55]. Regarding arrhythmia, emerging evidence suggests that SGLT2i can reduce the vulnerability of rats with right heart failure to ventricular arrhythmia via restoring calcium handling. Huang et al. found this involves increasing conduction velocity, the threshold for Ca^2+^ and APD alternans, and decreasing susceptibility to spatially discordant APD alternans and spontaneous Ca^2+^ events [14]. In addition, it is worth noting that these effects for calcium may be indirect and stem from SGLT2i’s effect on intracellular sodium. It requires a certain duration to manifest. A prolonged 24-hour exposure to SGLT2i has been demonstrated to reduce CaMKII activity and enhance calcium handling, whereas acute exposure does not affect CaMKII activity or calcium handling, but instead leads to a significant reduction in sodium current [55].

### Myocardial metabolism

The pleiotropic effects of SGLT2i for multiple organs (e.g. kidney, angiocarpy, brain, liver, gut) and multiple disease (e.g. DM, renal injury, HF, nonalcoholic fatty liver disease, inflammatory bowel disease, cognitive disorder) [77–80] suggest SGLT2i treatment may alter the systemic and myocardial metabolic state to achieve their beneficial effects, which have been well reviewed [81, 82]. And a growing number of evidences suggest that electrophysiologic derangements and arrhythmias are closely linked to changes in metabolic activity in a wide variety of pathological conditions [83]. Abnormalities in metabolism are also considered as the beginning of some arrhythmias, that precede electrophysiological, contractile and structural remodeling [84]. For instance, the total concentration of lipoprotein particles and the concentration of high density lipoprotein (HDL) particles were thought to mediate the inhibition of SGLT2i on AF, according to a mendelian randomization study [29]. Considering these, myocardial metabolism may be a key target for the antiarrhythmic effect of SGLT2i.

Constriction of cardiac muscle is a very energy demanding process and the heart can selectively utilize various energetic substrates. This selectivity will be changed with development and pathology [85]. SGLT2i effects for metabolic substrates have been investigated, which may switch myocardial fuel utilization to improve heart muscle energy metabolism as well as heart function. In non-diabetic animal models with HF and MI, empagliflozin improved myocardial energetics and cardiac ATP production, which benefited cardiac function and remodeling. And this effect was ascribed to empagliflozin shifting myocardial fuel use from glucose to ketone bodies, free fatty acid, and branched chain amino acids [86, 87]. Worthily, transcriptomics and metabolomics studies reported that a cardiac energy metabolism switch from fatty metabolism to glycolysis in AF patients [88]. Therefore, SGLT2i may improve AF by potentially altering the substrate utilization, despite the absence of direct evidence. However, two clinical studies of myocardial energy metabolism in T2D patients showed that empagliflozin did not improve myocardial energy metabolism during the 12-week empagliflozin treatment, although SGLT2i altered many cardiometabolic indicators (uric acid, high-density lipoprotein cholesterol, ketone bodies, and insulin sensitivity). Meanwhile, the same result appeared in high-fat-high-sucrose diet mice [17]. Although SGLT2i plays a certain role in myocardial energy metabolism, it may not improve the energy metabolic disorders under special pathology (e.g. T2D and high-fat diet). Our explain for the paradox is that improvement in myocardial energy metabolism may be an additional effect of SGLT2i in regulating metabolism, and this effect may be covered by metabolic disorders.

Another important hypothesis about the metabolism effects of SGLT2i suggests that it can cause special transcriptional and metabolic paradigm, which was reported as hypoxia and fasting-like transcriptional and metabolic paradigm that is characterized by loss of calories in the urine, decrease of plasma glucose and insulin, fuel utilization switch from carbohydrate to fat substrates, promotion of gluconeogenesis and ketogenesis, increased erythropoietin and erythrocytosis [45, 89, 90]. Sugar loss in urinate leads the decrease of plasma glucose and insulin levels. Body and fat mass loss can ascribe to SGLT2i leading to a fuel utilization shift from carbohydrate to fat substrates, that has been investigated in human and animal studies [91, 92]. In addition, the ketonemia and erythrocytosis caused by SGLT2i can be explained by the activation of the sirtuin 1/hypoxia-inducible factor 2 alpha (SIRT1/HIF-2α) pathway and upregulation of HIF-1α. SGLT2i upregulates the nutrient deprivation sensors SIRT1, that promotes gluconeogenesis and fatty acid oxidation as well as activates the rate-limiting step in ketone body synthesis [90, 93, 94]. Meanwhile, as a hypoxia-inducible factor that is the main driver of erythropoietin synthesis and erythrocytosis, HIF-2α can be activated by SIRT1 [95, 96]. The higher expression of HIF-1α was founded in empagliflozin and dapagliflozin treatment [97, 98]. Interestingly, Molecular docking results indicated the possibility of direct interaction with SGLT2i and SIRT1 [99]. Some metabolic changes themselves are beneficial for cardiovascular disease. Ketone bodies not only supply energy but also resist oxidative and inflammatory injury [100]. Ex vivo experiments with macrophages verified that SGLT2i modulates NLRP3 inflammasome activity via ketones and insulin to make cardioprotective effects [101]. Animal experiments also demonstrated empagliflozin-mediated elevation of cardiac ketones could ameliorate symptoms of heart failure with preserved ejection fraction (HFpEF) by preserving mitochondrial function and attenuating inflammasome activation [102]. More importantly, in this paradigm, SGLT2i improves the metabolic status of the whole body, including various target organs, causing changes in the corresponding molecules including AMPK, SIRT1, HIF, AKT and so on, which in turn play important functions in cellular autophagy, mitochondrial function, inflammation, oxidative stress and ferroptosis. All of these factors play a crucial role in the pathological mechanisms underlying arrhythmias and have the potential to serve as therapeutic targets.

### Autophagy

SGLT2i lead to hypoxia and fasting-like transcriptional and metabolic changes along with alteration of molecules (AMPK, SIRT1, HIF, AKT, et al.). These changes induce autophagy, a lysosome-mediated degradation pathway maintaining cellular homeostasis by removing harmful components (e.g. misfolded proteins, protein aggregates, unhealthy mitochondria and peroxisomes) and holding ATP levels during food deficiency [103]. Parallelly, autophagy reduces oxidative and endoplasmic reticular stress, and suppresses inflammasome activation and NFκB-mediated inflammation, by processing dysfunctional mitochondria and other organelles [104–106]. For example, SGLT2i enhance autophagy and elevate removal of damaged mitochondria in renal proximal tubular cells of obese mice, thereby reducing oxidative stress and inflammation in these cells [105]. Further, autophagy relates cell stress and inflammation regulate metabolism [107, 108]. Autophagy dysregulation causes metabolic disorders like insulin resistance, diabetes, obesity, atherosclerosis, and osteoporosis [108]. Thus, we propose SGLT2i alleviate autophagy defects and related oxidative stress and inflammation by triggering hypoxia and fasting-like transcriptional and metabolic changes and corresponding molecular alterations, affecting metabolism and creating a virtuous cycle (Fig. 2).

Autophagy deficiency causes oxidative stress, energy disorders and cardiomyocyte dysfunction and death. Excessive autophagy causes cell death, termed autosis, with autophagosome and autolysosome accumulation, organelle loss and nuclear convolution [109]. Thus, autophagy is a “double-edged sword”, especially in cardiovascular disease. For example, in myocardial ischemia and reperfusion, autophagy protects the myocardium during ischemia by ensuring energy use, engulfing damaged mitochondria and reducing oxidative stress, triggered by AMPK activation and mammalian target of rapamycin (mTOR) inhibition [103]. However, during reperfusion, significant autosis from excessive autophagy depending on *Beclin1* is observed, independently of AMPK, with increased ROS. Reducing autophagy relieves reperfusion injury in mice with systemic heterozygous Beclin1 deletion [109, 110].

Alterations in autophagy in arrhythmias are found. AMPK-dependent autophagy is found in atrial myocytes of rapid atrial pacing-induced experimental AF canines and chronic AF/paroxysmal AF patients [111, 112]. During reperfusion in fibrillated mouse hearts, Beclin1 and LC3B-II increase, as cellular autophagy markers [113]. Excessive autophagy associates with arrhythmia susceptibility after myocardial hypertrophy and MI [114, 115]. Inhibiting autophagy decreases arrhythmias in myocardial hypertrophy and MI. In hereditary cardiomyopathies causing arrhythmias, autophagy markers like LC3 and SQSTM1/p62 and autophagic vacuoles are detected in arrhythmogenic cardiomyopathy and LAMP2 cardiomyopathy [116, 117]. Excessive autophagy significantly promotes AF by degrading L-type calcium channels, shortening action potential duration and reducing L-type calcium current [118]. However, in another study, decreased LC3B-II and significant autophagic vesicle and lipofuscin deposit accumulation were found in postoperative AF patients [119]. Meanwhile, activating autophagy mitigated fibrosis and atrial remodeling in isoprenaline-induced AF [120]. These findings suggest autophagy deficiencies, impaired autophagic flux and autophagosome accumulation may also contribute to arrhythmia development. Heterogeneity of autophagy in arrhythmias may vary by underlying cause and mechanism, needing further research. Correcting autophagy disorders in arrhythmias may be a potential therapeutic target.

Excitingly, in other cardiovascular diseases, SGLT2i may optimize autophagy through multi-effect actions, regardless of autophagy absence or excess. SGLT2i stimulate autophagy to protect the heart and kidneys by inducing hypoxia and fasting-like transcriptional and metabolic changes, involving AMPK, SIRT1 and HIF activation and mTOR suppression [45, 121, 122]. Conversely, SGLT2i can suppress excessive autophagy to protect the heart through the NHE1/Beclin1 pathway in MI [43, 54].

AMPK senses ATP/AMP ratios, activating autophagy in response to cellular energy cues by inhibiting rapamycin complex 1 (mTORC1) and phosphorylating ULK1 [123]. Via the AMPK/mTOR/autophagy pathway, SGLT2i attenuate various diseases in animal models like cardiac dysfunction, diabetic cardiomyopathy, non-alcoholic fatty liver disease, acute kidney injury, cerebellar/neurobehavioral deficits [79, 124–128]. However, SGLT2i inhibit excessive autophagy via the AMPK/GSK3β pathway to alleviate diabetic cardiomyopathy [129]. And, dependently on AMPK, dapagliflozin attenuated increased NHE1 mRNA in lipopolysaccharide-exposed cardiofibroblasts [130]. NHE1 inhibition alleviates excessive autophagy, noted above [54]. This shows AMPK’s bidirectional ability to regulate autophagy, partly corresponding to SGLT2i’s two-way regulatory capacity.

SIRT1, an NAD-dependent redox-sensitive enzyme, regulates autophagy by deacetylating target proteins. SIRT1 directly interacts with and deacetylates several ATG proteins like Atg5, Atg7 and Atg8 [131]. It’s worth noting that SIRT1 regulates Beclin1, verified in SIRT1 knockout mice [132]. Therefore SGLT2i may upregulate SIRT1 and downstream mediators like PGC-1a and FGF21 through direct SIRT1 binding, activating autophagy in the heart [133]. SIRT1 also enhances insulin signaling and activates AKT at multiple levels [133]. AKT signaling inhibits autophagy through mTOR stimulation, possibly another SGLT2i bidirectional autophagy regulation mechanism [123].

Hypoxia-inducible factors, HIF-1α and HIF-2α, mediate cellular oxygen sensing and response, like autophagy [134]. Although both activate in hypoxia, they exert mutually antagonistic effects and balance. Imbalances between HIF-1α and HIF-2α may contribute to chronic heart failure, atherosclerotic and hypertensive vascular disorders progression [135]. HIF-1α upregulates autophagy through downstream molecules like BNIP3 and Beclin1, protecting in myocardial ischemia-reperfusion injury and hypoxic/ischemic brain injury [136]. SGLT2i activate HIF-1α and suppress HIF-2α to activate autophagy by causing hypoxia and fasting-like transcriptional and metabolic changes [97, 98, 137]. However, in other studies, SGLT2i had the opposite effect [130, 138]. Packer et al. proposed that SGLT2i suppressing HIF-1α and activating HIF-2α induced erythropoiesis and alleviate organelle dysfunction, inflammation and fibrosis [139]. The inconsistent findings suggest SGLT2i’s impact on HIF-1α and HIF-2α may be indirect, co-mediated by SIRT1 or AMPK or other undiscovered mechanisms. Nevertheless, SGLT2i can alleviate cardiac pathologies by optimizing HIF-1α/HIF-2α balance, like their dual-way autophagy effect. Further research is needed to better understand the mechanisms.

AKT is a serine/threonine kinase that plays a crucial role in the PI3K signaling pathway, which serves as a major mediator of insulin signaling. The AKT protein exerts its effects by activating the mechanistic target of mTORC1 and suppressing autophagic gene expression. The actions of AKT can be countered by the AMPK [140]. Via the PI3K-AKT-mTOR pathway, inhibiting autophagy alleviates arrhythmias in myocardial hypertrophy or MI [114, 115]. While the impact of SGLT2i on AKT has been observed in many studies, there is no recorded association with arrhythmias [141–143]. 

### Intersection of two main pathways

SGLT2i may alter multiple cardiac ion channels through metabolic improvement of transcriptional regulation. AMPK affects cardiac ion channels through various mechanisms to regulate cardiac Na-Ca homeostasis [84]. Sodium ion channels may be a substrate of AMPK. Studies have shown that high activity of AMPK leads to a significant prolongation of action potential duration and early afterdepolarizations that can cause potentially arrhythmias. High activity of AMPK significantly slows down the inactivation of Nav 1.5 and shifts the voltage activation curve towards hyperpolarization [144]. AMPK activation can increase the rate of intracellular Ca^2^ + clearance, restore calcium transients and contractility reduction, related to increased activity of NCX and Cav1.2 channels. In addition, AMPK can regulate HCN4, that regulates encoding the funny current, Kir2.1 and Na-K-ATPase [84, 145]. The PI3K/AKT signaling pathway is also a key regulatory node for multiple cardiac ion channels, regulating Nav1.5, Kv11.1, Kv7.1 and Cav1.2 channels in cardiomyocytes [146]. Many anti-cancer drugs that inhibit PI3K have been shown to prolong APD and QT interval through multiple mechanisms such as increasing INa,L [147]. Cardiomyocytes intracellular dialysis with PIP3 normalizes ion channel alterations and eliminates proarrhythmic features [148].

SGLT2i possibly coordinate NHE1, AKT interactions and jointly regulates autophagy. Activation of AKT is often coupled with inhibition of NHE1. Besides the function of effecting intracellular sodium, NHE1 was reported to be crucial in regulating autophagy [149]. In the research of Xiang et al., NHE1 induced the excessive autophagy after myocardial infarction (MI). Empagliflozin treatment could inhibit NHE1 to suppress cardiomyocytes autophagic cell death to confer cardioprotective effects, like reducing infarct size and myocardial fibrosis, improving cardiac function and survival. Cardioprotective and downregulation of autophagic flux in NHE1 knockout mice after MI demonstrated that NHE1 exerts a cardiovascular protective effect at least in part through regulating autophagy [54]. The activation of AKT can suppress NHE1, and inhibiting AKT can correspondingly enhance NHE1 [150, 151]. Snabaitis et al. suggested that NHE1 is a substrate of AKT, whose phosphorylation at Ser648 site mediated by AKT during cell acidosis interferes with CaM binding and reduces affinity for intracellular hydrion, inhibiting myocardial layer NHE1 activity [152]. However, degradation of NHE1 would also activate AKT, preserving insulin sensitivity and altering substrate processing of mouse heart and whole-body metabolism [70]. Therefore, NHE1 inhibition by SGLT2i may suppress autophagy either through AKT activation or by regulating pH, inhibiting relocation of lysosomes to cell edges and secretion of Cat B [153]. At the same time, SGLT2i may also regulate autophagy by improving metabolic activation of AKT. Autophagy can regulate NHE1 expression by affecting NRF2 transcription, relevant to the intracellular sodium pathway discussed earlier [154]. Upregulation of NRF2 pathway is also thought to mediate the anti-oxidative stress and anti-AF effects of dapagliflozin therapy in myocardial injury induced by lipopolysaccharide [155].The sequence of which, NHE1 or AKT, plays a leading role still needs further study. We speculate that this order has a certain temporal and spatial specificity.

SGLT2i-mediated autophagy optimization and transcriptional changes may mitigate Na-Ca imbalance-induced arrhythmia through ROS/CaMKII pathways. Conversely, SGLT2i-mediated sodium load relief may come from alleviating oxidative stress and inflammation in arrhythmia through CaMKII/ROS pathways [55]. Increased ROS, CaMKII activation, and disruption of Na-Ca homeostasis in myocardial cells are characteristic of various arrhythmias. The three factors promote each other to form a vicious cycle that contributes arrhythmia and heart dysfunction [156]. On the one hand, SGLT2i reduce ROS by optimizing autophagy pathways such as mitochondrial autophagy [105]. On the other hand, SGLT2i activate the AMPK pathway and its downstream molecules such as PGC-1α, NRF2, TGF-β, and SMAD to reduce ROS, promote mitochondrial remodeling, inhibit mitochondrial fission, reduce inflammation and energy expenditure, and regulate ferroptosis [93, 157–160]. These effects may operate independently of their role in regulating autophagy. SGLT2i-mediated ROS reduction can reverse CaMKII activation, improve Na-Ca imbalance, and further exert anti-arrhythmic effects. In addition, SGLT2i reduces intracellular sodium and improves calcium overload, which reduces CaMKII activation and improves arrhythmia matrix (e.g. inflammation and fibrosis) [161].

### Other mechanisms

In the aforementioned text, we have emphasized that the distinctive transcriptional and metabolic paradigm induced by SGLT2i can bidirectionally regulate autophagy through the corresponding molecular modifications, regardless of the presence of deficient or excessive autophagy. These modifications may have effects beyond merely regulating autophagy and contribute to the anti-arrhythmic properties of SGLT2i in other pathways. For instance, Kadosaka et al. suggested the impact of SGLT2i on calcium handling may be due to reducing O-GlcNAcylation through inhibiting glucose uptake, not NHE1 inhibition [162]. The AKT/eNOS/NO pathway can ameliorate endothelial damage, which could be a result of SGLT2i suppressing NHE1 [163, 164]. Meanwhile, SGLT2i reduces the activation of NLRP3 inflammasome, which plays an important role in the development mechanism of cardiac arrhythmia [165]. The improvement in autonomic activity, decrease in body weight and blood pressure, and reduction in fat accumulation may also partly contribute to the anti-arrhythmic effects [166].

## Conclusion

As previously indicated, we have conducted a comprehensive summary of the existing clinical data on the antiarrhythmic effects of SGLT2i involving the prognosis and incidence of arrhythmias, and established a new paradigm to investigate the potential mechanisms of SGLT2i in mitigating arrhythmias. This paradigm suggests that SGLT2i impact intracellular sodium levels through its actions on Nav1.5 (regulating INa,L) and NHE1. Meanwhile, they evoke the transcriptional and metabolic paradigm resembling hypoxia and fasting and optimizes autophagy, accompanied by alterations in multiple molecular entities, such as AMPK, SIRT1, HIF, and AKT. These two pathways complement and cooperate with each other to promote the antiarrhythmic effects of SGLT2i. Among them, Nav1.5, NHE1, and SIRT1 may exert their effects by directly binding with SGLT2i. Based on existing clues, we speculate that the first pathway (intracellular sodium) plays an immediate role, dominating ion channel function in the short term and regulating intracellular sodium-calcium homeostasis. The second pathway (metabolism-autophagy) plays a long-term role and dominates in prolonged treatment. However, the new paradigm we propose is essentially an experimental framework that incorporates as much evidence as possible. In the pathway mechanism from SGLT2i to the treatment of arrhythmias, many associations are supported only by indirect evidence. And there are still numerous gaps waiting to be filled or refined by future evidence. Furthermore, this new paradigm may have implications for studying the effects of SGLT2i in the field of heart failure, as the antiarrhythmic effects and anti-heart failure effects of SGLT2i are interconnected rather than two distinct and parallel phenomena. In conclusion, SGLT2i exert multi-layered impacts through various pathways and has significant implications for various cardiovascular diseases especially arrhythmias. Nevertheless, further research is warranted to reveal the complex mechanisms and other physiological effects behind the therapeutic benefits of SGLT2i.

### Electronic supplementary material

Below is the link to the electronic supplementary material.

Supplementary Material 1

## Data Availability

Not applicable. No new datasets were generated for this review article.

## Abbreviations

SGLT2i: Sodium-glucose cotransporter type 2 inhibitors
HF: Heart failure
T2D: Type 2 diabetes
VT/VF: Ventricular tachycardia/ventricular fibrillation
AF: Atrial fibrillation
VAs: Ventricular arrhythmias
DM: Diabetes mellitus
CKD: Chronic kidney disease
AFL: Atrial flutter
AMI: Acute myocardial infarction
SCD: sudden cardiac death
HFrEF: Heart failure with reduced ejection fraction
HFpEF: Heart failure with preserved ejection fraction
NHE1: Sodium-hydrogen exchanger 1
INa,L: Late sodium channel current
INa,P: Peak sodium current
CaMKII: Calmodulin-dependent kinase II
DAD: Delayed afterdepolarization
EAD: Early afterdepolarizations
ROS: Reactive oxygen species
AMPK: AMP-activated protein kinase
NLRP3: Nuclear-binding domain-like receptor 3
APD: Action potential duration
HDL: High density lipoprotein
SIRT1: Sirtuin 1
HIF: Hypoxia-inducible factor
mTOR: Mammalian target of rapamycin
