# Supplementary material for: The potential anti-arrhythmic effect of SGLT2 inhibitors
Source: Cardiovasc Diabetol. 2024 Jul 15;23:252. doi: 10.1186/s12933-024-02312-0 (PMC11251349; doi:10.1186/s12933-024-02312-0)
Supplement: Supplementary file 1 — Supplementary Material 1 [file 12933_2024_2312_MOESM1_ESM.docx]

**Supplementary material**

**The Potential Anti-Arrhythmic Effect of SGLT2 Inhibitors**

**Running** **title:** *Duan, et al. - Anti-Arrhythmic Effect of SGLT2 Inhibitors*

**Hong-Yi Duan, MDa,b,c,** **Hector Barajas-Martinez, PhD, FAHA, FHRSd,e,** **Charles Antzelevitch, PhD, FAHA, FACC, FHRS d,e, Dan Hu,** **MD, PhD, FAHA, FACC, FHRSa,b,c,***

**a.** Department of Cardiology, Renmin Hospital of Wuhan University, Wuhan, 430060, China;

**b.** Cardiovascular Research Institute of Wuhan University , Wuhan, 430060, China;

**c.** Hubei Key Laboratory of Cardiology, Wuhan, 430060, China.

**d.** Lankenau Institute for Medical Research, and Lankenau Heart Institute, Wynnwood, Pennsylvania, 19096, USA

**e.** Sidney Kimmel Medical College, Thomas Jefferson University, Philadelphia, 19107, USA

***Correspondence should be addressed to**

**Dan Hu,** MD, PhD, Professor

FAHA, FACC, FHRS, FAPHRS

Specialty Chief Editor of *Frontiers in Cardiovascular Medicine*

Department of Cardiology & Cardiovascular Research Institute,

Renmin Hospital of Wuhan University,

238 Jiefang Road, Wuhan, 430060, China.

Email: hudan0716@hotmail.com, or rm002646@whu.edu.cn

Phone: 86-27-88041911

Fax: 86-27-88042292

**ORCID: 0000-0002-0994-8434**

**Twitter: @hudan0716**

| **Supplemental Table S1. Meta-Analyses on the Improvement of Arrhythmia with SGLT2i** | | | | | | |
| --- | --- | --- | --- | --- | --- | --- |
| **Drug** | **No. of Patients** | **Population** | | **Main findings** | **Refs.** | |
| **Arrhythmia Prognosis** | | | | | | |
| Sotagliflozin (2)  Empagliflozin | 3 RCTs  2469 patients | DM+AF | Composite of HF hospitalization and Cardiovascular death ↓ (HR 0.70) | | | (1) |
| **Atrial Arrhythmias** | | | | | | |
| Canagliflozin (6)  Dapagliflozin (8)  Empagliflozin (7)  Ertugliflozin (1) | 22 RCTs  29,211 SGLT2i vs 22,904 placebos | DM, CKD, HF | AF ↓ (RR 0.82)  Embolic stroke ↓ (RR 0.32)  AF/AFL ↓ (RR 0.82)  VT ↓ (RR 0.73) | | | (2) |
| Various SGLT2i | 6 RCTs  4,731 SGLT2i vs 4,736 placebos | HFrEF | AF ↓ (RR 0.62)  AF/AFL ↓ (RR 0.64) | | | (3) |
| Dapagliflozin (11)  Canagliflozin (10)  Empagliflozin (9)  Ertugliflozin (4) | 34 RCTs  35,883 SGLT2i vs 27,273 control | T2D | Atrial arrhythmias ↓ (OR 0.81)  SCD ↓ (OR 0.72) | | | (4) |
| Dapagliflozin (7)  Canagliflozin (7)  Empagliflozin (5)  Ertugliflozin (1) | 20 RCTs  63,604 patients | Majority T2D, minority HF and CDK | AF ↓ (OR 0.82) | | | (5) |
| Various SGLT2i | 8 RCTs  31,261 SGLT2i vs 24,705 control | ±DM | AF ↓ with and without diabetes (RR 0.79) | | | (6) |
| Canagliflozin (6)  Dapagliflozin (11)  Empagliflozin (8)  Ertugliflozin (4)  Sotagliflozin (2) | 31 RCTs  75,279 patients | Majority T2D, minority HF, CDK and high cardiovascular risk | Serious AF ↓ (RR 0.75) | | | (1) |
| Empagliflozin (2)  Canagliflozin (3)  Dapagliflozin (3)  Ertugliflozin (1) | 9 RCTs  high dose: 22,525 SGLT2i vs 22,508 no SGLT2i  low dose: 10,599 SGLT2i vs 10579 no SGLT2i | Majority T2D,  minority HF, CDK and high cardiovascular risk | High dose or low dose  AF ↓ (RR 0.79)  Bradycardia ↓ (RR 0.60)  Cardiac failure acute ↓ (RR 0.68)  Cardiac failure chronic ↓ (RR 0.75)  NO significantly different between high-dose and low dose | | | (7) |
| Dapagliflozin (6)  Empagliflozin (4)  Canaglifozin (6) | 16 RCTs  38,335 patients | T2D | AF/AFL ↓ (RR 0.76)  All-cause mortality ↓ (RR 0.91) | | | (8) |
| Canagliflozin | 14543 patients | T2D, High risk of cardiovascular disease, CKD | In those with no AF/AFL history  AF/AFL ↓ (HR 0.78)  AF/AFL-related complications ↓ (ischaemic stroke/transient ischaemic attack/hospitalization for HF) (HR 0.74) | | | (9) |
| Ertugliflozin (2)  Canagliflozin (8)  Dapagliflozin (5)  Empagliflozin (7) | 22 RCTs  29,658 SGLT2i vs 23,317 no-SGLT2i | T2D or HF | In T2D/HF patients  AF/AFL ↓ (OR 0.82),  Arrhythmia ↓ (OR 0.86)  Intracardiac thrombosis ↓ (OR 0.31)  In T2D patients  AF/AFL ↓ (OR 0.80),  In women  AF/AFL ↓ (OR 0.83)  Dapagliflozin  AF/AFL ↓ (OR =0.85),  Intracardiac thrombosis ↓ (OR 0.31) | | | (10) |
| Dapagliflozin (4)  Empagliflozin (3)  Ertugliflozin (1) | 8 trials  19,236 SGLT2i vs 19,271 no-SGLT2i | ± DM | AF ↓ (pOR 0.76)  Dapagliflozin, AF↓ (pOR 0.73)  > 1 year follow-up, AF ↓ (pOR 0.75)  History of cardiovascular disease or CVRF, AF ↓ (pOR 0.80)  T2D, AF ↓ (pOR 0.80) | | | (11) |
| Canagliflozin (2)  Dapagliflozin (2) | 4 RCTs  CANVAS, CREDENCE, DECLARE-TIMI 58 and DAPA-HF  32,535 patients | T2D, high risk of cardiovascular disease, CKD, HFrEF | AF/AFL ↓ (HR 0.81)  History of AF/AFL, AF/AFL ↓ (HR 0.80) | | | (9) |
| **Ventricular Arrhythmias** | | | | | | |
| Canagliflozin (6)  Dapagliflozin (8)  Empagliflozin (7)  Ertugliflozin (1) | 22 RCTs  29,211 SGLT2i vs 22,904 placebos | DM, CKD, HF | AF ↓ (RR 0.82)  Embolic stroke ↓ (RR 0.32)  AF/AFL ↓ (RR 0.82)  VT ↓ (RR 0.73) | | | (2) |
| Empagliflozin (6)  Canagliflozin (6)  Dapagliflozin (7) | 19 RCTs  55,590 patients | T2D, HF, CKD | low-dosage SGLT2i therapy  VAs ↓ compared to control (RR 0.45)  VAs ↓ compared to placebo (RR 0.46) | | | (3) |
| Note: SGLT2i: sodium-glucose co-transporter 2 inhibitors; RCT: randomized controlled trial; T2D: type 2 diabetes; DM: diabetes mellitus; CV: cardiovascular; AF: atrial fibrillation; AFL: atrial flutter; VA: ventricular arrhythmia; VT: ventricular tachycardia; VF: ventricular fibrillation; HF: heart failure; AMI: acute myocardial infarction; CKD: chronic kidney disease; HFrEF: heart failure with reduced ejection fraction; TIA: transient ischemic attack; SCD: sudden cardiac death; OR: odds ratio; HR: hazard ratio; RR: relative risk; aHR: adjusted hazard ratio; pOR: partial odds ratio. | | | | | | |

**References**

1. Pandey AK, Okaj I, Kaur H, Belley-Cote EP, Wang J, Oraii A, et al. Sodium-Glucose Co-Transporter Inhibitors and Atrial Fibrillation: A Systematic Review and Meta-Analysis of Randomized Controlled Trials. Journal of the American Heart Association. 2021;10(17):e022222.

2. Li HL, Lip GYH, Feng Q, Fei Y, Tse YK, Wu MZ, et al. Sodium-glucose cotransporter 2 inhibitors (SGLT2i) and cardiac arrhythmias: a systematic review and meta-analysis. Cardiovascular diabetology. 2021;20(1):100.

3. Sfairopoulos D, Liu T, Zhang N, Tse G, Bazoukis G, Letsas K, et al. Association between sodium-glucose cotransporter-2 inhibitors and incident atrial fibrillation/atrial flutter in heart failure patients with reduced ejection fraction: a meta-analysis of randomized controlled trials. Heart failure reviews. 2023;28(4):925-36.

4. Fernandes GC, Fernandes A, Cardoso R, Penalver J, Knijnik L, Mitrani RD, et al. Association of SGLT2 inhibitors with arrhythmias and sudden cardiac death in patients with type 2 diabetes or heart failure: A meta-analysis of 34 randomized controlled trials. Heart rhythm. 2021;18(7):1098-105.

5. Zheng RJ, Wang Y, Tang JN, Duan JY, Yuan MY, Zhang JY. Association of SGLT2 Inhibitors With Risk of Atrial Fibrillation and Stroke in Patients With and Without Type 2 Diabetes: A Systemic Review and Meta-Analysis of Randomized Controlled Trials. Journal of cardiovascular pharmacology. 2022;79(2):e145-e52.

6. Okunrintemi V, Mishriky BM, Powell JR, Cummings DM. Sodium-glucose co-transporter-2 inhibitors and atrial fibrillation in the cardiovascular and renal outcome trials. Diabetes, obesity & metabolism. 2021;23(1):276-80.

7. Zou HT, Yang GH, Cai YJ, Chen H, Zheng XQ, Hu R. Are High- or Low-dose SGLT2 Inhibitors Associated With Cardiovascular and Respiratory Adverse Events? A Meta-analysis. Journal of cardiovascular pharmacology. 2022;79(5):655-62.

8. Li WJ, Chen XQ, Xu LL, Li YQ, Luo BH. SGLT2 inhibitors and atrial fibrillation in type 2 diabetes: a systematic review with meta-analysis of 16 randomized controlled trials. Cardiovascular diabetology. 2020;19(1):130.

9. Li C, Yu J, Hockham C, Perkovic V, Neuen BL, Badve SV, et al. Canagliflozin and atrial fibrillation in type 2 diabetes mellitus: A secondary analysis from the CANVAS Program and CREDENCE trial and meta-analysis. Diabetes, obesity & metabolism. 2022;24(10):1927-38.

10. Wang M, Zhang Y, Wang Z, Liu D, Mao S, Liang B. The effectiveness of SGLT2 inhibitor in the incidence of atrial fibrillation/atrial flutter in patients with type 2 diabetes mellitus/heart failure: a systematic review and meta-analysis. Journal of thoracic disease. 2022;14(5):1620-37.

11. Ong HT, Teo YH, Teo YN, Syn NL, Wee CF, Leong S, et al. Effects of Sodium/Glucose Cotransporter Inhibitors on Atrial Fibrillation and Stroke: A Meta-Analysis. Journal of stroke and cerebrovascular diseases : the official journal of National Stroke Association. 2022;31(1):106159.
